# The quality of life after a total gastrectomy with extended lymphadenectomy and omega type oesophagojejunostomy for gastric adenocarcinoma without distant metastases

**DOI:** 10.1186/1471-2482-12-11

**Published:** 2012-06-27

**Authors:** Gintare Jakstaite, Narimantas Evaldas Samalavicius, Giedre Smailyte, Raimundas Lunevicius

**Affiliations:** 1 Clinic of Oncosurgery of Oncology Institute, Clinic of Internal Diseases, Family Medicine and Oncology of Medical Faculty, Vilnius University, 1 Santariskiu Street, Vilnius, LT-08660, Lithuania; 2 Liver, Renal & Surgery Department, King’s College Hospital NHS Foundation Trust, King’s Health Partners Academic Health Sciences Centre, Denmark Hill, London, SE5 9RS, UK

**Keywords:** Gastric cancer, Total gastrectomy, Extended lymphadenectomy, Omega esophagojejunostomy, Quality of life

## Abstract

**Background:**

To evaluate the quality of life (QOL) in relation to age, sex, clinical stage, postoperative complication, and adjuvant chemotherapy in patients who underwent curative total gastrectomy with D2 lymphadenectomy and Omega type esophagojejunostomy for gastric adenocarcinoma.

**Methods:**

69 patients were included. Lithuanian version of the European Organization for Research and Treatment of Cancer Quality of Life Questionnaire Cancer 30 was sent to all of them from six months to two years after gastric surgery for self-completion. 34 questionnaires were filled and were used as material for further analysis. Influence of age (≥ 65 *vs* < 65), sex, clinical stage (I–II *vs* III), surgical complication, and adjuvant chemotherapy was assessed on QOL in this retrospective cross-sectional case series study.

**Results:**

The global health status was better in the group of patients aged over 65 (63.0 points *vs* 46.4, P = 0.0509). The functional scales were higher in the same group of patients. Significant difference was only observed on the social scale in favour of elders (P = 0.0039). Sex, clinical stage, surgical complications, and postoperative chemotherapy had no significant influence on any aspect of QOL.

**Conclusion:**

The global QOL and the social functioning was better in patients aged 65 years and over, compared to patients under the age of 65 in the period of 6 to 18 months after a total gastrectomy with D2 lymphadenectomy and Omega esophagojejunostomy.

## Background

Improving cancer therapy leads to increasing survival rates. In addition to this, there is more attention being placed on the quality of life (QOL) which is mostly dependent on cancer diagnosis and complex treatment [1]. Thorough assessment on the QOL of patients is especially important when surgery is applied as a main option of treatment, as the operated patients often suffer from various functional and psychological symptoms for a considerably longer time than the average amount of months following the surgery. These symptoms and other aspects of health which necessitate for changes of lifestyle, have to be analysed by investigating the QOL.

QOL is a multidimensional construct which represents comfort and well-being of the patients, secondary to the disease and treatment [2]. Research has shown that patients want information not only about the treatment outcomes but also about the influence of treatment on their lifestyle [3]. This is the reason why the information about the QOL should become a part of fully informed consent taking procedure before surgical treatment [4]. On the other hand, when physicians consider QOL as one of the points of prognosis after particular surgical procedure, they often rely solely on their personal professional experience because there is not much formal data about QOL except for few locations of cancer, for instance, breast or prostate [5]. The rationale for this research was the fact that little attention, if any, has been focused on QOL after extended curative surgery for gastric cancer with particular type of gastrointestinal continuity reconstruction. The aim of this retrospective cross-sectional study was, therefore, to evaluate the QOL in relation to age, sex, clinical stage, postoperative complication, and adjuvant chemotherapy in patients who underwent curative total gastrectomy with D2 lymphadenectomy and Omega type esophagojejunostomy for gastric adenocarcinoma without distant metastases.

## Methods

The curative R0 total gastrectomy for middle or/and proximal gastric adenocarcinoma with reconstruction of digestive tract by means of an esophagojejunostomy with a jejunal loop and Braun’s side-to-side enteroanastomosis was performed on 87 patients in the Institute of Oncology of the Vilnius University, Lithuania, from January 2008 to July 2009. All specimens were evaluated histologically. Gastric adenocarcinoma was staged according to 7th edition of TNM classification of malignant tumours [6]. An extended lymphadenectomy D2 was based on principles described and developed by Japanese gastric cancer association [7]. 69 patients, who were without distant metastasis or proven recurrence 6 to 18 months after surgery, were included into the retrospective cross-sectional study. None of these patients received neoadjuvant treatment prior to surgery.

The Lithuanian version of the European Organisation for Research and Treatment of Cancer Quality of Life Questionnaire Cancer 30 (EORTC QLQ-C30) was used to assess the QOL of gastric cancer patients and sent to them for self-completion in January 2010. 36 patients (52.2%) had responded. 34 questionnaires were filled thoroughly and they were used as material for further investigation.

The main characteristics of those patients are shown in the Table 1. The influence of age (≥ 65 years vs. < 65), sex, clinical stage (I–II *vs* III), surgical complication (yes vs. no), and adjuvant chemotherapy (yes vs. no) was assessed on QOL. All data are expressed as mean ± using standard deviation. Quality of life scores were compared between groups using the Mann–Whitney *U*-test. Differences with a P value of 0.05 were considered to be statistically significant. Microsoft Office XP Excel 2007 Worksheets were used for accumulation and analysis of data.

**Table 1:** Characteristics of 34 patients who filled the EORTC QLQ-C30

**Characteristics**	**Value/patients**	**Per cent**
Age Median	64	–
Range	42-84	–
SD	10.91	–
≥ 65	18	52.9
< 65	16	47.1
Sex		
Male	20	58.8
Female	14	41.2
Stage of gastric cancer		
I	5	14.7
II	12	35.3
III	17	50.0
Histology		
Well-differentiated adenocarcinoma	1	3.0
Moderately differentiated adenocarcinoma	8	23.4
Poorly-differentiated adenocarcinoma	25	73.6
Postoperative complication		
Yes	7	20.6
No	27	79.4
Adjuvant chemotherapy		
Yes	16	47.1
No	18	52.9

## Results

**Figure 1:**
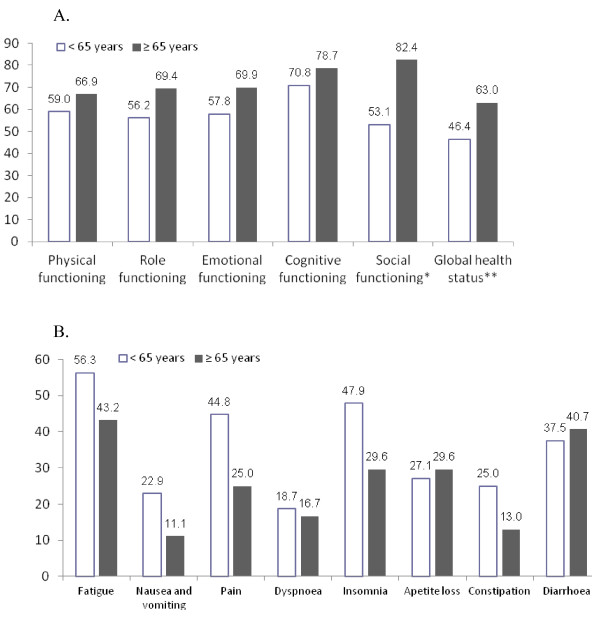
Impact of age on the quality of life at time of total gastrectomy with extended lymphadenectomy and Omega type esophagojejunostomy (* P = 0.0039, ** P = 0.0509, Mann–Whitney* U* test).

The age of patients had an obvious tendency to influence the global health status (Figure 1A). It was better in the group of patients aged 65 years and over (63.0 points *vs* 46.4 points, P = 0.0509). The functional scales were also higher in the group of patients aged 65 and over. The significant difference was only observed on the social scale and the global outcome showed in favour of elders (82.4 vs. 53.2, P = 0.0039). Symptoms, especially pain (44.8 vs. 25.0) and insomnia (47.9 *vs* 29.6), were more common in patients under age 65. However, the differences between these groups were not significant (Figure 1B).

**Figure 2:**
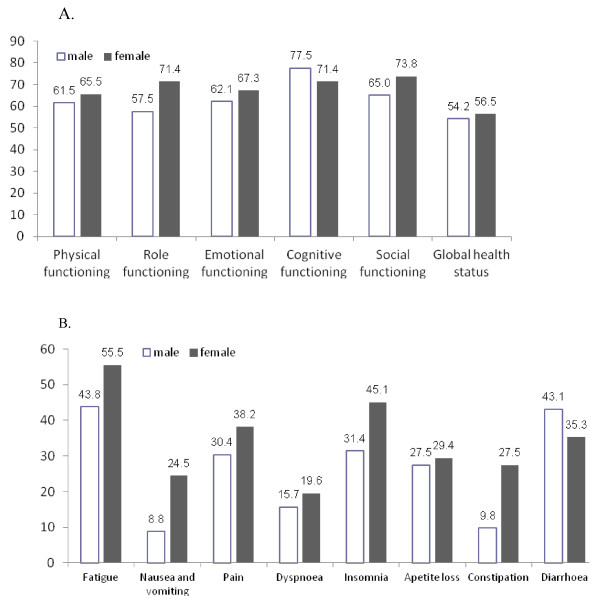
Influence of sex on the quality of life at time of total gastrectomy with extended lymphadenectomy and Omega type esophagojejunostomy (no significant differences).

Analysis of the influence of sex on the global health status (54.2 vs. 56.5) and functional scales have shown no differences between male and female (Figure 2A). Although symptoms like nausea and vomiting, insomnia, constipation were more often complained by woman (24.5 vs. 8.8, 45.1 *vs* 31.4, 27.5 vs. 9.8, respectively), a statistical significant difference was not found (Figure 2B).

**Figure 3:**
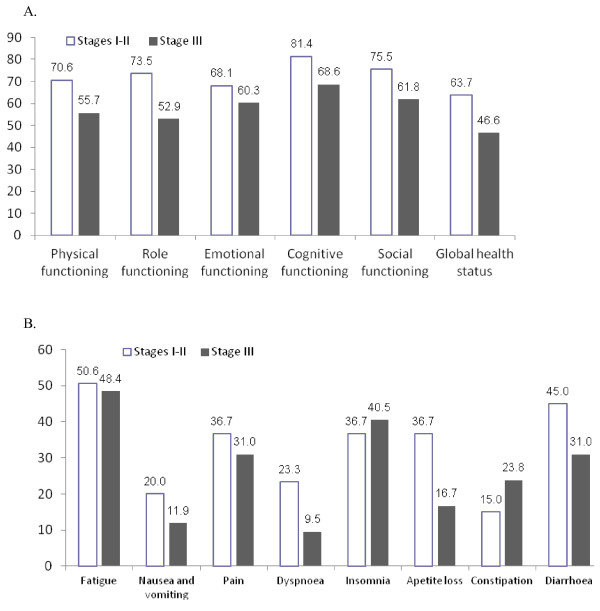
Impact of clinical stage of the gastric cancer on the quality of life at time of total gastrectomy with extended lymphadenectomy and Omega type esophagojejunostomy (no significant differences).

The global health status (63.7 vs. 44.6) and functional scales were higher in patients with I and II clinical stages for gastric adenocarcinoma (Figure 3A). On the other hand, these patients expressed more symptoms such as fatigue, nausea and vomiting, pain, dyspnoea, loss of appetite, and diarrhoea (Figure 3B). Nevertheless, differences between groups were not statistical significant.

Neither statistical significant differences between groups nor obvious trends, an analysis of surgical complications and adjuvant chemotherapy on QOL were revealed.

## Discussion

The studies on the QOL of gastric cancer patients mostly feature the direct interview and evaluation of patients’ symptoms or performance status by physician [5], although the evaluation of QOL is more valuable when it is expressed by the patients themselves [8]. Also, any study on QOL should cover as many aspects of life as possible. Kaptein et al. wrote a review on all QOL studies of patients with gastric cancer which showed that all the studies included physiological aspects but none of them included social functioning [5]. As the biopsychosocial model of medicine is increasingly becoming significantly important, the wider spectrum QOL research is becoming increasingly valuable [5,9].

The EORTC QLQ-C30 is an extensively tested questionnaire on cancer patients which can be used to evaluate the QOL of cancer patients in any country. Furthermore, it is a combination of adequate psychometric characteristics which give an opportunity of comparing between patients with different categories of cancer [10]. The same questionnaire was used in the studies which analysed QOL of cancer patients who followed radical surgical procedures as well [10-13]. The EORTC QLQ-C30 consists of 30 items which ask how the patient would rate his or her health and all the aspects associated with it during the last week [14]. Every item belongs to a different scale or it is a single-item measure. There are five functional scales, three symptom scales, a global health status, and six single-items in the questionnaire. All of the scales and single-item measures have been transformed linearly, ranging from 0 to 100. The data was evaluated by the guidelines of the EORTC [10,11]. It is important to note that higher scores for the functional scales and the global health status reflect better quality of life, while high scores for the symptom scales represent problems which influence the QOL negatively.

Total curative gastrectomy consists of two phases: the removal of the stomach with a limited or extended lymphadenectomy and the reconstruction of the gastrointestinal tract. Nowadays, the most popular method of restoration of continuity of the gastrointestinal tract is Roux-en-Y one [8]. This might be the reason why so little information on outcomes of other reconstructions as well as Omega technique can be found. The importance of knowledge on postoperative QOL as it may be an important factor in clinical decision-making, including considering surgery or not in a subgroup of patients with limited life expectancy was emphasized [15].

Several studies analysed a relation between QOL and clinical, demographic, and social parameters as it was done in this study [14,16-20]. Analysis of global health care status of our patients has shown, surprisingly, that the elder patients scored a better QOL in comparison to those younger, and is especially noticeable in social functioning. It is not simple to explain this finding of the study as other similar studies which pointed out a different trend – younger patients had better QOL after gastric surgery than older [19,20]. In our view, the fact that the global QOL and the social functioning was better in Lithuanian patients aged 65 years and over may be related with a less demanding, and, therefore, slightly more positive outlook to disease burden and surrounding environment. Nevertheless, one should be cautious in interpreting key finding of this study because of nature of this study. On the other hand, de Liaño et al. also indicated that older patients’ global health status was better, however, he also found that older patients had more clinical symptoms which is a contrasts to our study [14].

Physicians often link the advanced stage of cancer with the poorer quality of life. Although the scales of the earlier-stage cases were a little bit higher, this study as well as Huang et al. study [18] had not shown any significant differences between early and advanced cancer stages when at least 6 month had passed after surgical treatment. On the other hand, Matsushita et al. found that patients in the later stage of gastric cancer had a significantly worse quality of life [17]. Furthermore, there is no single opinion about the influence of postoperative complications following total gastrectomy on QOL. In addition to our study, de Liano et al. stated that QOL had no relation with postoperative complications. However, Matsushita et al. concluded that it negatively influences the physical functioning of gastrectomized patients after 6 months [14,17]. We have to note that women had higher scores almost in all functional and symptom’s scales dimensions. Nevertheless, there were no statistically significant differences. Again, data are controversial regarding gender role on QOL [14,19,21].

They are few limitations of this study. It is a retrospective study whereby there were a small number of patients responders involved; it undoubtedly caused a bias view. There was no control group. In addition to this, as the QOL is a subjective feature, it is influenced by not only diagnosis or treatment, but by the character and psychological state of the patients as well [17].

## Conclusions

Data of the study show that the global QOL and the social functioning was better in patients aged 65 years and over, compared to patients under age 65 in the period of 6 to 18 months after a total gastrectomy with extended lymphadenectomy and Omega esophagojejunostomy for gastric adenocarcinoma without distant metastases and recurrence. In our view, this study delineated a question for further research related to type of curative surgery for elderly and survivorship. It, as a retrospective cross-sectional study of QOL after total gastrectomy with Omega reconstruction of gastrointestinal tract, can provide background for design of both retrospective case–control and prospective randomised clinical studies. Meanwhile, results of previous and current studies that include QOL in patients with gastric cancer should be applied in preoperative as well as postoperative clinical care, which aims at improving the QOL of these patients after the total gastrectomy with particular method of gastrointestinal reconstruction.

## Competing interests

The authors declare that they have no competing interests.

## Authors’ contribution

NES and RL equally participated in the design of the study, critical revision, and definitive drafting. GJ carried out the accumulation of the data, literature review, and provisional drafting. GS carried out the statistical analysis of data. All authors read and approved the final manuscript.

## Pre-publication history

The pre-publication history for this paper can be accessed here:

http://www.biomedcentral.com/1471-2482/12/11/prepub
